# When someone has low vision

**Published:** 2012

**Authors:** Clare Gilbert, Karin van Dijk

**Affiliations:** Co-director, International Centre for Eye Health, London School of Hygiene and Tropical Medicine, Keppel Street, London WC1E 7HT, UK; Clinical Advisor, Sightsavers; CBM global advisor on low vision; low vision consultant to Light for the World Netherlands and to Kilimanjaro Centre for Community Ophthalmology. Grutto 21, 7423CZ Deventer, The Netherlands, kvdijknl@yahoo.com

**Figure:**
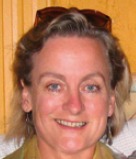
Clare Gilbert

**Figure:**
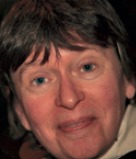
Karin van Dijk

As clinicians, being faced with a patient whose vision we cannot improve any further can make us feel like a failure.

However, there are many ways to help such a person with low vision.

Figure 1 shows the difficulties someone is likely to have, based on their distance visual acuity, and what support they may be able to benefit from. These include optical devices, non-optical devices, advice on environmental modifications, and referral to rehabilitation and (special) educational services.

In this article, we will show you how to assess a person with low vision and find out what it is they really want to be able to do. We will then outline the interventions that are possible, and give some guidelines.

## Before you start

When you are faced with a person with poor vision, it is important to check that everything possible has been done to improve their vision, and that they really do need low vision services. Here is a checklist:

1. Has the person's diagnosis been confirmed by an ophthalmologist or other eye care worker?
  **Figure:**
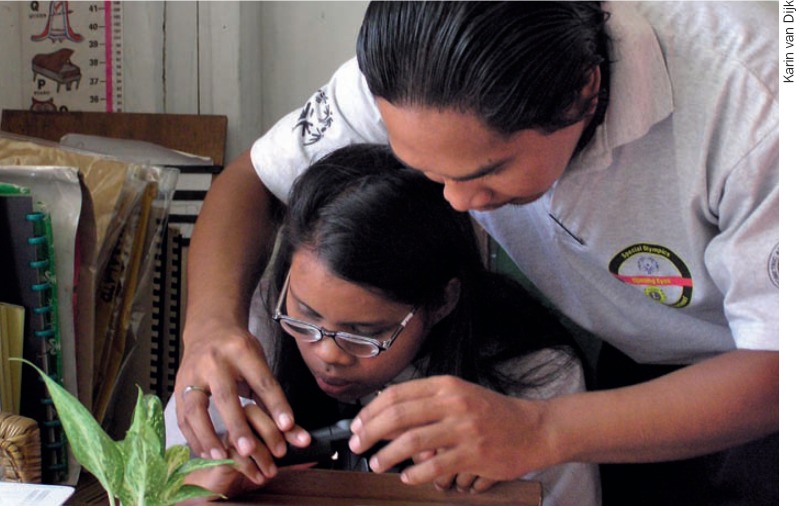
Teaching the use of an illuminated hand magnifier. PHILLIPINES
2. Has all the medical, surgical, and optical treatment possible already been given?
3. Has the prognosis for vision been confirmed by a medical professional?

If the answer to any of these questions is ‘no’, refer the person to the appropriate services, where possible.

If we know the diagnosis, this will give us some idea of the likely impact on the person's visual function and thus on their main visual needs (see page 2).

Ideally, people with low vision should have undergone refraction, and be wearing their spectacles, before they are given low vision support. In practice, many eye care practitioners find it too challenging and/or time-consuming to refract someone with low vision. This is why refraction should always form part of a standard low vision assessment.

Once you have established that the person does need low vision services, you can begin the low vision assessment.

The following are the steps that normally form part of a low vision assessment:

- Taking a history
- Explaining the eye condition
- Determining the patient's needs
- Performing an accurate refraction
- Assessing visual functions
- Magnification needed
- Designing a management plan
- Referral for further training and support and contacting educational or rehabilitation services if needed.
- Selecting low vision devices and training the person in their use
- Suggesting non-optical interventions and environmental modifications.

**Figure 1.**
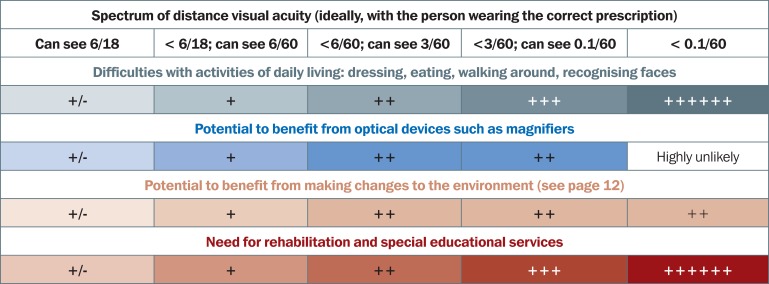
How the type of assistance provided is influenced by distance visual acuity

## Taking a history

This is an important part of the low vision assessment and provides an opportunity for you and the patient to get to know each other.

Encourage the patient to talk about their problems. Asking open-ended questions will help; these are questions starting with words such as ‘when’, ‘what’, ‘how’, and ‘where’ – questions which don't have ‘yes’ or ‘no’ as an answer.

Ask questions about:

- Their own eye health – how their vision is affected, what makes it worse or better and how it has changed over time
- Their general medical history, their mobility, and their medications
- Their family's eye health history
- Their occupation and hobbies
- Any previous low vision assessments.

Here are examples of questions you can ask about their eye health and vision:

- When did you first notice a problem with your vision?
- What kinds of problems have you noticed?
- What problems do you have in the day?
- What problems do you have at night?
- What changes, if any, have you noticed in your vision?
- What makes your vision worse?
- What makes your vision better?

## Explaining the eye condition

Some people with low vision will not have had their eye condition explained to them, or they may not have understood the explanation at the time.

It is always worthwhile taking time to explain the eye condition again, in terms the person can understand. Even if patients with low vision have heard it all before, they will probably find it reassuring to have you explain it again, thereby confirming what they have heard from others.

Be positive. Emphasise that they have some residual vision and that you and your colleagues are committed to helping them make the most of that vision. Reassure them that they cannot harm their residual vision by using it – they will **not** ‘wear out’ their eyes!

## Determining the patient's needs

Start on a positive note by first asking what they can still do, before going on to ask what they may be struggling with.

Ask about their mobility, activities, and participation. Here are some examples.

### Mobility

- Can you walk beyond the house without assistance?
  **CASE STUDY 1**
  *This case study, and those that follow, are of actual people and demonstrate practical low vision assessments and interventions. They show how the diagnosis and history can guide us in setting priorities for assessment and knowing which interventions, especially non-optical, might benefit the person*.
  **A 60-year old retired professor** with age-related macular degeneration complained that he could no longer read small text, which had been an important part of his life. He also taught college students and worked extensively on the computer at home. On further questioning, it became clear that he also had difficulties in communicating with others. From the history, interview, and diagnosis, we knew that the man had central field loss and reduced contrast sensitivity, which would require improved lighting and contrast.
  The low vision team assessed his best corrected distance and near visual acuity, contrast sensitivity, reading and writing ability, and the extent of his field loss.
  His visual acuity, tested on a logMAR chart, was 6/36 (0.8 logMAR) in the better eye, and with a +2.00D add his near vision was 1M (N8) at 15 cm. His near acuity improved to 0.63M at 25 cm with an add of +3.00D, a reading lamp, and a reading slit. With these, he was also able to read the newspaper and his writing was legible.
  The professor was advised to wear his bifocal glasses constantly, to read with a table lamp and reading slit, and to use a reading stand. A signature guide helped him to sign cheques.
  He was taught how to use eccentric viewing (see page 8), which helped him to recognise people more easily. This helped him socially.
  The professor was advised about the importance of explaining to his friends and family why he was notable to make direct eye contact.
  He was also directed to the local government office to obtain a disability certificate and other paperwork.
- Can you walk around in familiar places without assistance?

### Activities

- Can you choose and find the clothes you want to wear?
- Can you add the correct spices and herbs to the food while cooking?
- Can you still do your hobby, e.g., needlework or woodcarving?
- Can you read religious texts, the newspaper, or utility bills?

### Participation

- Do you attend family functions?
- Do you attend religious or other events?
- Are you still able to vote?

Check with relatives that this is what they have observed or experienced; sometimes people feel embarrassed to acknowledge how dependent they have become.

It is also important to find out what kind of support they have at home.

- Who do they live with, and is this person able to provide help some of the time, or all of the time?
- Is providing this support having a negative effect on the family in anyway?
- What is the home like? Are there steps? Where are the washing and sanitation facilities? How is cooking done?

Having established broadly what support they have at home, and what they can and cannot do in relation to mobility, activities, and participation, find out what they want to be able to do. This will guide the interventions you suggest.

Ask for specific examples of what would help them to regain independence or self esteem. For example:

- Regaining the ability to read their personal correspondence
- Helping to cook again instead of just sitting around
- Learning to identify the correct medication and taking it independently
- Making a visit to a neighbour on their own, whenever they feel like it.

When discussing these topics, think about the following:

- Do they need help with near and intermediate vision, with distance vision, orwith all distances?
- Is the task long (reading) or short (looking at the oven temperature dial)?
- Do they need to have one or both hands free?
- What other visual functions might be affected and must be assessed?

## Accurate refraction

The importance of good refraction in a low vision assessment cannot be overstated.

Refracting people with low vision differs from refracting people whose vision can be improved to normal (6/6 or 20/20), as the person with low vision is less sensitive to small changes in the power of trial lenses and may respond much more slowly. Patience is essential, and using the bracketing technique (see panel) can help.

**Figure 2.**
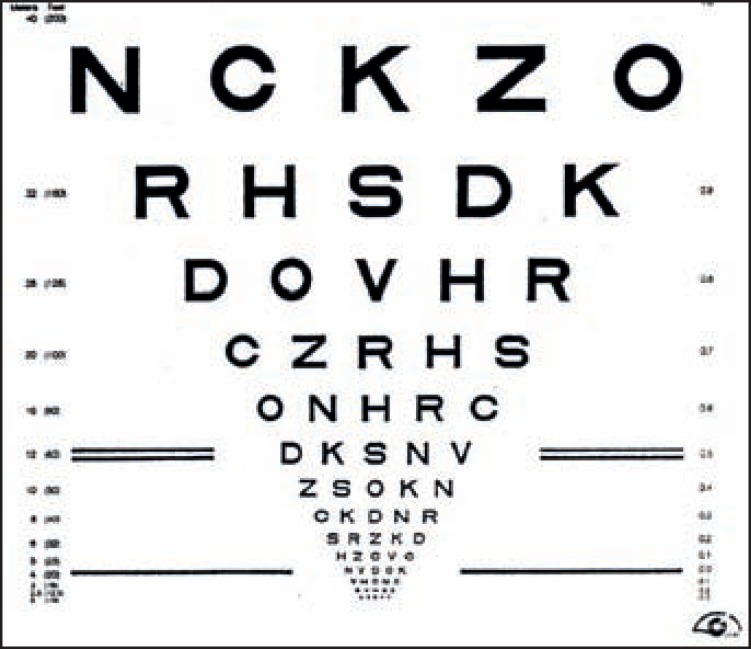
A log MAR chart has an equal number of letters in every line, regular spacing between lines and letters, and a uniform progression in letter size

Fatigue and frustration can negatively influence the outcome of the refraction. Ensure the person is seated comfortably and give them time to recover from any signs of stress or tiredness.

While doing refraction, the test chart should be at a distance where the patient can see at least the top line of letters. Full aperture trial lenses should be used to allow the patient to move their head or eyes in order to fixate eccentrically (see panel on page 8).

Assess the near addition (lens) needed and measure the working distance with which the patient is comfortable. Record their best corrected near and distance visual acuity.

## Assessing residual vision

The support we provide depends on having a thorough understanding of the person's overall visual function. For example, people with poor contrast sensitivity may require more magnification than suggested by their near visual acuity alone.

When assessing someone with low vision we therefore need to have a better idea of their overall visual function, including:

- Distance visual acuity
- Near visual acuity
- Contrast sensitivity
- Visual fields
- Light sensitivity
- Colour vision

If you work in a setting with limited resources, the improvement of distance and near visual acuity can be emphasised; the other visual functions can be tested functionally, as suggested here.

If you work at a large eye hospital, use the appropriate tests and equipment.

### Distance visual acuity

We are used to testing distance visual acuity using standard Snellen charts at only two distances: six metres (20 feet) or three metres. However, when testing someone with low vision, we should preferably use logMAR charts as they give better measures of acuity. If the person cannot see the letters at three metres, we must also test at other test distances, such as two metres, one metre, etc.

**CASE STUDY 2**

**45 year-old man with A glaucoma**, who drove himself to work in a factory, was referred to the low vision clinic. He was married with two school-age children, and was the main breadwinner in the family.

The man said that he had problems with driving and working in the evenings, and had difficulty navigating inside the factory. These difficulties were the result of loss of peripheral field and reduced contrast sensitivity related to the glaucoma. He also had difficulty in crossing roads, identifying curb edges, walking in shaded places, and identifying landmarks. These findings suggested a need for better illumination in the evenings and in situations of poor lighting.

The low vision assessment included distance and near visual acuity with best correction, visual field testing by confrontation, and contrast sensitivity testing using light-coloured objects against a dark background. Mobility was tested in different lighting conditions by going for a short walk with the client. His distance visual acuity was 6/24 (0.60 logMAR) with his myopic glasses of-4.00D. He could read IM (N8) without his glasses at 20 cm.

A 6D hand-held magnifier was prescribed to make reading the newspaper and small print on the machinery more comfortable, and he was advised to wear a cap with a visor to reduce glare when in bright sunlight. After consultation with his employer, levels of illumination in the factory were increased. This improved contrast, enabling him to navigate doorways and concrete pillars more easily. This improved his mobility, working efficiency and confidence. He was advised to travel to work using public transport or share rides with co-workers. The need for regular review and continued use of glaucoma medication was explained.

**Using the bracketing technique**

Because patients with poor visual acuity may have difficulty in determining small changes in lens power and clarity, it is often necessary to make large power changes.

A bracketing technique can be useful. For example, use a +2.00DS trial lens and compare this with a −2.00DS trial lens. If the patient is able to differentiate between the two, the lens giving the better vision can be added and the technique repeated using, say, a −1.00DS and a −1.00DS lens, etc. It may be necessary to refract the patient with the chart placed at 3 m or less. If this is done, the result is over-plussed (the chart at 3 m acts as a near object and therefore adds a vergence of about −0.30D in the plane of the trial lens) and a correction should be made according to the test distance.

Adapted from: Subjective Refraction: Principles and Techniques for the Correction of Spherical Ametropia, Andrew Franlin http://www.banjoben.com/low_vision_refraction.htm

### Near visual acuity

It is very important to test everyone's near vision, not just those who can read and write, as good near vision is needed for a very wide range of other activities. We must also know the near visual acuity so that we can prescribe low vision magnifiers for near tasks, if needed.

Near visual acuity can be tested using logMAR charts (Figure 2) similar to those used for testing distance visual acuity. It is important that comparable tests for both are used. The choice of test depends on age, development level, and literacy of the client, e.g., tumbling Es or Landolt rings.

It may be useful to assess near vision at a distance of 25 cm (see article on page 9). Note that people with presbyopia may need an appropriate addition in order to read at this distance. In addition to near vision, reading and writing performance should be assessed among those who are literate. This is because reading requires other functions that are not assessed in acuity testing, for example, locating the next line of print. If near acuity only is measured, difficulties with reading may be missed.

The best way to assess reading is to use printed text from a newspaper or book and to ask the person to read it aloud. Reading aloud allows the assessor to hear mistakes and observe the person's visual skills.

### Contrast sensitivity

Contrast sensitivity is the measure of the eye's ability to detect differences in greyness and background, or small changes in brightness. Most of our world is in moderate to poor contrast. Visual acuity charts are one of the few things in high contrast!

Reduced contrast sensitivity can be assessed functionally by asking questions such as:

- Do you find it more difficult to walk around in very bright sunlight, or at dawn and dusk?
- Can you see the white light switch on the light-coloured wall in your house?
- Can you read your bills (which are often on grayish paper, with poor contrast)?

There are several ways of testing contrast sensitivity clinically, such as the Pelli Robson chart, but these charts are expensive and require that the person with low vision is literate. A less expensive alternative is the Lea low-contrast flip chart (see page 13 for ordering details), which is suitable for those who are not literate, including children. Low contrast may explain why a person with a visual acuity of 6/36 can manage many tasks well, but struggles in poor light.

#### Contrast: tips for daily activities

It is not easy to translate these findings to impact on daily activities. In general, moderate contrast sensitivity might have an impact on reading, whereas very poor contrast sensitivity might indicate the need for visual rehabilitation and mobility training.

You can help people with low contrast sensitivity by advising them how to increase contrast in their environment. There are two main ways:

1. **Use better lighting**. For example, sit by the window to read or sew, or use a lamp. Be aware: very bright light, including direct sunlight, can reduce contrast.
2. **Make adaptations in the environment**. For example, use paint or coloured tape to create contrasting strips on steps or around light switches.

### Light sensitivity

Both too little light, and too much light (glare), can affect what someone with low vision is able to see.

People with **increased light sensitivity** struggle to see in the presence of bright light (for example, light reflected by a shiny blackboard or table top). This is a common problem for people with low vision.

In the presence of such bright light, or glare, contrast is reduced and recognisin objects or people can become very difficult.

**CASE STUDY 3**

**Diabetic retinopathy made a 75-year-old woman** unsure of her bearings at home, even though she had undergone cataract surgery with intra-ocular lens implantation. She was unable to identify different utensils and other items, such as spices, in the kitchen. She also could not see the knobs on the gas cooker. She was keen to do her own cooking, gardening, reading, and shopping.

Pseudophakia is accompanied by loss of accommodation, while diabetic retinopathy can result in sensitivity to light, patchy field loss, with reduced contrast sensitivity and color discrimination.

These visual functions were all assessed. Her best corrected distance visual acuity was 6/60 (1.0 logMAR) with astigmatic correction.

With a near add of +4.00D, she could read 1.6M at 20 cm.

The interventions recommended focused on improving her near visual acuity and included an 8 dioptre illuminated stand magnifier, which enabled her to read IM print, using a reading stand to help her read more comfortably. She could also use the magnifier to identify money.

She was trained in the use of eccentric viewing to assist in daily activities and was shown how to fold paper money in different ways so she could tell which amount they were for.

To help in the kitchen she was advised to use different coloured labels for different pots and to use containers of varying shapes or sizes for her spices. She was also advised to remove all un necessary furniture in the living areas. Other non-optical interventions she liked were a signature guide for banking, and extra illumination for near work.

People with **reduced light sensitivity** also struggle to see, and will often also have reduced contrast sensitivity.

#### Light: tips for daily activities

Getting the amount of light right is the key intervention in this situation. Ask what the person is struggling with, such as seeing at night (reduced light sensitivity) or seeing outside in bright sunlight or when the light reflects off the blackboard (increased light sensitivity).

For people with reduced light sensitivity, recommend that they sit near a window or try different lamps.

**Figure 3.**
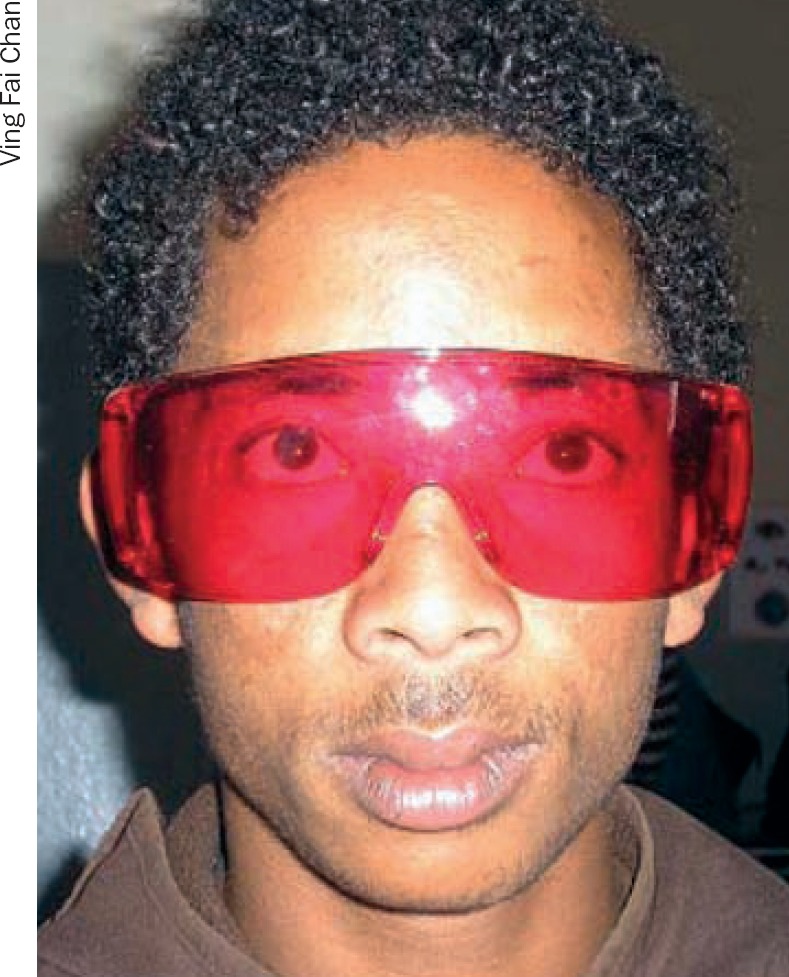
A red filter has helped this man with achromatopsia (a rare form of colour blindness causing extreme light sensitivity) to see in daylight

You can determine the best lighting conditions for particular tasks, such as reading or sewing, by letting the person try out different types of lamps in the clinic.

People with increased light sensitivity could wear tinted glasses, sunglasses, or a cap outdoors to help with glare.

Filters (Figure 3) can help people with both contrast and/or light sensitivity by minimising glare and increasing contrast. Filters look like safety glasses and are available at low cost (see page 13 for ordering details). Many different colours and shades are available, such as yellow, brown, grey, red, etc.

People may need two different shades of a particular filter: one for indoor use (light) and one for outdoor use (dark).

## Visual fields

Ideally, the clinician making the diagnosis will have assessed the patient's visual fields as part of their clinical assessment. If not, questions can help.

Patients may realise that they cannot see detail clearly but can see well enough to walk around. This suggests central visual field loss; this is often due to macular degeneration.

Someone with peripheral field loss from glaucoma or retinitis pigmentosa can see detail but will bump into furniture or fall over things on the floor.

There are a range of tests available, including confrontation (face-to-face) testing, static tests (e.g. Friedmann visual field analyser), and dynamic tests (e.g. tangent screen or Goldmann tests).

The Amsler grid test is used to plot areas of significant visual loss within the central 20° of the visual field (the area of the retina providing fine detail). The person is tested while wearing their reading glasses or bifocals, if appropriate.

For perimetry, e.g. Humphrey perimetry, a new hand-held perimeter is available from the Low Vision Resource Centre (see page 13). It is quick to use and provides reliable and repeatable results.

### Visual fields: tips for daily activities

For people with central visual field loss:

1. Provide high magnification
2. Show them how to use eccentric viewing (see panel below).

For peripheral visual field loss, the best advice is to keep pathways clear and to avoid moving furniture in the house. A cane for walking around outside may be very useful.

## Colour vision

It is rare fora person to be completely colour-blind, but reduced colour vision occurs more often in people with low vision. This can be assessed by asking questions such as: do you have difficulty when trying to find clothes of matching or similar colours? Have you noticed any problems when discriminating shades of colours?

There are formal methods for colour vision testing, such as Ishihara plates and the Farnsworth dichotomous test (D-15), which involves colour arrangement. In practice, it is usually sufficient to see

**Eccentric viewing**

It is likely that people with loss of central vision (often associated with macular degeneration) will need to develop an eccentric viewing technique, in which they use their peripheral vision instead of their central vision. They might find it easier to see things if they do not look directly at them, but rather to one side or the other.

Eccentric viewing can be difficult to teach, and to learn. However, you could start by encouraging the person to try finding the best area for viewing for themselves, starting with real objects, then faces, and later on with larger letters or words. The person will eventually learn to control their eye movements.

If you have internet access, you can visit http://www.mdsupport.org/evtraining.html fora guided introduction in how to use eccentric viewing.

**Figure 4.**
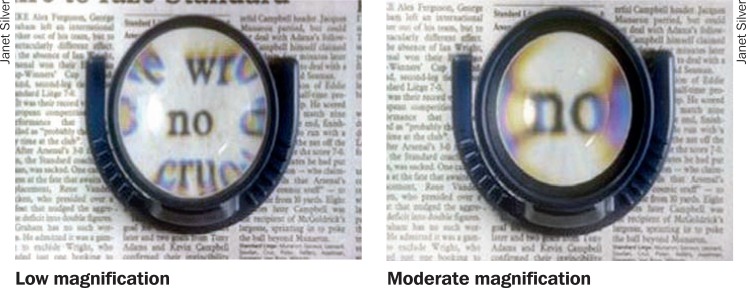
Increasing magnification reduces the field of view (right)

whether the person can see or match the primary colours, e.g. red, green, and blue. This can be tested using pencils or pieces of coloured fabric, for example, and asking the person what colour they see.

However, clinical colour vision testing can be valuable to make the correct diagnosis concerning the cause of a person's decreased vision.

### Colour: tips for daily activities

People with a colour vision deficiency or with blurred vision may find it difficult to distinguish between two colours that are similar. Suggest the following:

- Arrange the food cupboard so tins or foods of contrasting colours are next to each other
- Ask someone to help label clothes or to put matching outfits together ahead of time (on the same hanger/shelf)
- Use other senses (touch and smell) to find out which fruit are ripe.

## Magnification needed

Many people with low vision can benefit from magnification: using lenses to make objects appear bigger. However, magnification has its limitations. It is important to understand these limitations and explain them to the people you are helping so they have realistic expectations about what is possible.

- Stronger magnifiers have smaller lenses. You cannot have a strong magnifier that has a big lens!
- Stronger magnifiers have more distortion around the edge of the lens, which means you can see clearly through the centre of the lens only.

So, although the object or word looks bigger, only a few letters or a small part of the object can be seen at any one time (see Figure 4). This reduces reading or working speed.

Therefore, we recommend you prescribe the **lowest possible** power of magnifier that can be used comfortably fora longtime (if needed).

With electronic devices such as closed-circuit television cameras and electronic readers, the same limitations do not apply. However, these devices are a lot more expensive than lenses.

**Remember:** to maximise the benefit of magnifiers, it is important that people wear an up-to-date pair of distance correction spectacles when testing magnifiers and that they wear their reading spectacles with stand magnifiers.

For suggestions on predicting the level of near magnification someone will require, see the article opposite.

## Designing a management plan

Develop a management plan based on all the information you have gathered about the person with low vision.

Ask yourself: what does the person need? This depends on their history, their physical capabilities, the nature of their residual vision, and what they want to do. You may suggest some or all of the following:

- Optical low vision devices: for near or distance vision
- Non-optical interventions, such as caps for glare, a reading stand to reduce fatigue, a reading guide, various lamps, filters, sunglasses, etc. See the ‘tips’ given on pages 7 and 8; the case studies also contain useful ideas.
- Environmental modifications, such as painting lines on stairs or using contrasting colours around the home (see page 12 and the case studies in this article).

Think about when the person should come back to see you again. Make an appointment if possible.

This is also the point during the low vision assessment where you consider what other support the person will need, for example, educational support and/or visual rehabilitation and mobility training.

Write the necessary letters or notes and ensure the person knows where to go.

If possible, follow up with the referral service to check whether your patient has taken up the referral. If not, why not?

**Figure 5.**
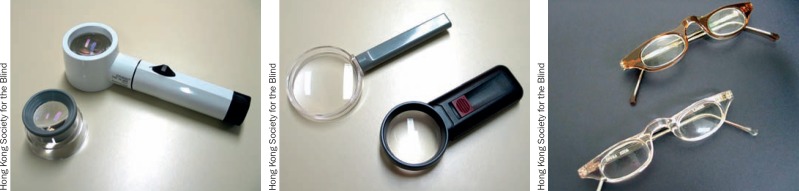
Examples of optical low vision devices (left to right): stand magnifiers, hand-held magnifiers, and spectacle magnifiers

## Selecting a low vision device

Start by thinking about the following:

- The person's visual abilities: can both eyes be used? Think about refractive error, ability to accommodate, and age
- The task the person wants to do: can one or both hands be free?
- The time for the task: short (such as checking a medicine label) or long (reading a story)? For a short task, a hand-held magnifier is fine, but for long periods of reading, dome, stand, or spectacle magnifiers would be better
- The physical condition of the person. If the person's hands tremble, a hand-held magnifier is not useful and a spectacle magnifier would be better.

Other considerations include:

- The availability of the device
- How acceptable it is
- How much it costs
- How much the person has to learn to use the device. Will the person come back if the device is difficult to use?

At the first appointment, try to focus on providing just one low vision device. Choose the easiest problem to solve, or the one that is most urgent for the patient.

**FROM THE FIELD: How to train people to use low vision devices**

**Figure:**
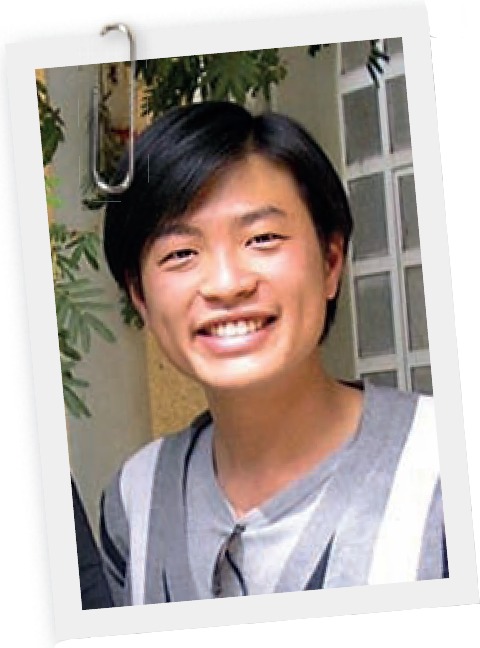
**Ving Fai Chan** is an optometrist who works for the International Centre for Eye Care Education (ICEE). He is a lecturer at the Asmara College of Health Sciences in Eritrea and is the only person providing low vision services in that country.

The first time I tried to drive a car, my dad was sitting beside me, expecting me to do it right the first time. When I struggled, I was extremely disappointed and felt I would never drive again.

It is the same when people with low vision try to use a device for the first time. We, as low vision practitioners, may expect them to know how to use the low vision devices perfectly, without giving them any encouragement or training. We think that, as long as the person has achieved his or her desired vision in the clinic, our job is done. Far from it!

Using low vision devices involves the development of completely new skills, often involving complex hand-eye coordination. And this requires practice. What seems natural and easy to us, such as focusing a telescope, feels quite unnatural to a patient the first time. The only way to solve this problem is to support and encourage our patients continuously.

Here are some basic steps:

1. Always explain to patients that it is fine if they are unable to perform the task the first time. Emphasise that this is normal.
2. Try to explain that there are things they can and cannot do as a result of their decreased vision, even with the help of the low vision devices. If that is not made clear, patients will have unrealistic expectations and will be disappointed with the results – which means they may give up learning how to use the device.
3. Give clear and step-by-step instructions. People with low vision usually respond well to verbal instructions. You can also give written instructions if the person or a family member is literate. Use good contrast and bigger letters where possible.
4. Provide regular, routine training. Teach your patients new skills only after they have mastered the previous ones. Giving too much information at once will make your patients feel stressed.
5. Follow up your patients. Everyone loves to be cared for. Encourage them and praise them when they have done well. Try to build their confidence and listen to their challenges. Sometimes it is better to listen than to talk.
6. Help them to solve their challenges one at a time. Sometimes meeting someone else with low vision can show patients that it is possible to overcome their problems.

Whenever my patients come back with a problem, providing support and encouragement is always the best way I can help them.

**‘Follow up your patients. Everyone loves to be cared for’**

**Figure:**
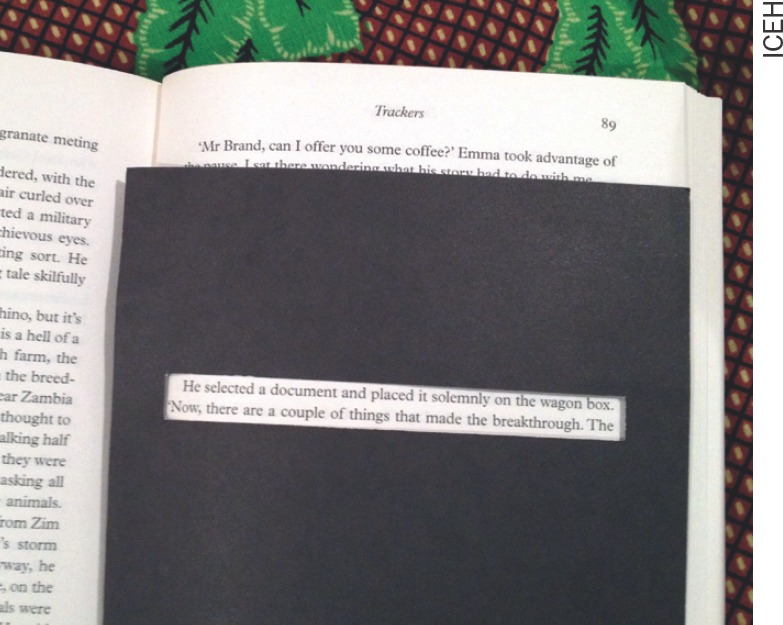
A reading guide or reading slit helps to improve contrast, and it may reduce the amount of magnification needed.

It takes time to learn how to use a new low vision device; learning one device successfully builds the person's confidence and they will be more likely to come back for further support.

Depending on the task the person wants to do, demonstrate one or more low vision devices that will provide the magnification they need. Allow them time to try the devices for themselves to see which work best.

Where possible, let them do something similar to what they would like to do at home, work, or school. Check the ease with which they are able to use the different devices and suggest modifications as needed. For example: add a reading guide, provide a reading stand, or increase available light.

### Adapting the magnification to fit the person

The magnification you predict a person will need (see page 9) is merely a starting point. Consider increasing the magnification by the smallest step possible for the following factors:

- Poor light: if there is no electricity or the light is dim and cannot be improved
- Tasks done for a longer time, such as reading or studying
- Poor contrast, such as bills or other printed matter with poor contrast
- A longer working distance needed, for example, if the person is physically unable to hold reading materials closer.

Demonstrate one or more devices that will provide the magnification the person needs to do their chosen tasks, and let them choose which one works best.

It is important to listen to the person: what is comfortable for them? What can they physically manage? There is no point in giving someone a magnifier which they don't enjoy using.

With thanks to Tanuja Britto (ophthalmologist) and Anitha Jayan (rehabilitation professional), Joseph Eye Hospital, Tiruchirapally, India

