# Supplementary material for: When someone has low vision
Source: Community Eye Health. 2012;25(77):4–11. (PMC3404439)
Supplement: Related texts

# Brief report: How to predict the near magnification needed

**Published:** 2012

It is possible to use a simple formula to predict the amount of near magnification a person might need.

The actual amount of magnification needed will vary according to the person's visual needs, environment, and the low vision device chosen. However, this is a useful starting point when selecting low vision devices to try out.

In this article, we use the testing distance of 25 cm, rather than the usual 40 cm, for two reasons:

1. Bringing objects closer makes them easier to see and improves contrast, important for people with low vision.
2. If we know the magnification needed at 25 cm, it is easy to calculate the dioptres needed to provide this.

## Finding the magnification needed for reading

The formula we use is given below. It lets us predict the amount of magnification the person will need (2x, 6x, etc.).

### Step 1. Find the near acuity achieved at 25 cm

- Make sure the person is wearing their distance prescription, if any
  **Figure:** Use a reading chart with sentences (or an E chart for people who cannot read). Hold it 25 cm from the face. VIETNAM
- Use a reading chart with sentences. Ask them to hold the chart at 25 cm
- If needed, particularly in older people, add positive lenses (from +1DS to +4DS) to both eyes, to enable them to accommodate at 25 cm
- Ask the person to read aloud
- The smallest size the person can read with comfort and good speed gives their near acuity at 25 cm (Note: this is not the smallest size they can see!)
- Write down the **near acuity achieved at 25 cm**, whether in M or N notation.

**Note:** M sizes differ depending on the testing distance used. Table 1 shows the M sizes of different types of text at both 25 cm and 40 cm. The latter is more familiar to most people. For our purposes, it doesn't matter which you use, provided you use the same notation throughout.

### Step 2. Find the required near acuity at 25 cm

- Ask what they want to be able to read
- Determine the text size and record the **required near acuity at 25 cm**, in the same notation.

**Note:** Don't aim to record the smallest size a person can see. Recording the size the person can read with comfort and speed in **Step 2** ensures that you will be able to choose the right amount of magnification.

### Step 3. Use the formula to calculate the magnification needed

- Divide the near acuity achieved at 25 cm by the required near acuity at 25 cm. This gives the amount of magnification required. **For example:** near acuity achieved is 2M, required near acuity is 1M: 2x magnification is needed to achieve this
- Now that we know the magnification needed, we can calculate which dioptre lenses can provide this level of magnification at 25 cm.
  **Figure:**
- So to provide 2x magnification at 25 cm (see example above), a device of 8 dioptres (D) is needed: 2 × 4 = 8D
- If you do not know the dioptres, check if the box of the device gives the ‘x’ (e.g., 2x) magnification. This is often given for a reference distance of 25 cm, which is the same distance used in this section.

**Table 1.** Text sizes in M notation (at both 25 and 40 cm) and in N notation

**M (40 cm)**	**M (25 cm)**	**N**	**Usual type text size**
2.0	3.2	16	Large print
1.6	2.5	12	Children's books
1.25	2.0	10	Magazine print
1.0	1.6	8	Newspaper print
0.8	1.25	6	Paperback print
0.6	1.0	4	Footnotes

## Magnification for tasks other than reading

Magnification is useful for many other tasks, not just reading. For example, sewing, sorting seeds, and drawing.

For people who cannot read, test their near vision using an E chart and record the size they can **very** easily see at 25 cm (Step 1). It is much easier to see the directions of just five ‘E’ letters than it is to read sentences, so the tester must resist the temptation to ask the client to read smaller and smaller sizes. Stop early!

Estimate a size of text equivalent to the level of detail they would require for their activity (Step 2). You can now predict the near magnification required (Step 3).

Use the actual activity they want to perform to try out the different magnifying devices, so keep sewing thread and needles, or seeds for sorting, etc., in the clinic; or ask the person to bring their materials. Advise them on different ways of holding their work and the magnifier, and give advice on lighting. If they need two hands free for their activity, spectacle magnifiers are the best device to use.

*With thanks to Karin van Dijk, Caroline Clarke, Mark Esbester, and Renee du Toit*.

# Brief report: How to work with children and older people

**Published:** 2012

It might take time and creativity to assess a young child, but with patience and appropriate tests distance and near visual acuity can be measured. Other visual functions such as contrast sensitivity can be assessed by asking the child and/or the parents. The following are also important:

- Children are more adaptable than adults and learn fast. They may be able to manage a range of devices for a range of activities, with some being used at school while others are used at home for play.
- Explain to parents it is good to use the remaining vision, even if the child sees less well than other children or only very little. Parents need to understand that spectacles and devices help the child to see better and use their vision better, and that these will not “weaken” their sight or damage their child's eyes.
- It is useful to find out what beliefs people have about eye conditions in the local community so that eye care workers know what to ask about. For example, people might believe that children with albinism should not be touched, or that the use of spectacles is a sign that vision is deteriorating, or that children who are born with poor vision cannot be helped.
- Many children with low vision have had poor vision since birth and will have developed their own way of learning. They need support to explain to others, especially their peers, why they cannot see everything, what they can and cannot do easily using their vision, and why they might need to use devices such as magnifiers or telescopes. One of the reasons children with low vision do not do well in school is that they fear the negative reactions of peers and/or teachers.

Children with low vision should ideally be re-assessed at least every year. Ensuring they come for follow-up is an important task of the low vision service. This is because:

- The demand on vision increases as they get older (e.g., the size of text in books becomes smaller in higher grades)
- As children grow, they will need bigger spectacle frames; their prescription is also likely to change every year
- Children are more likely to break, scratch, or lose their spectacles.

Give a written summary of all findings and recommendations to the parents and staff at school. In addition, explain the following in clear and simple language:

- The practical implications of the eye disease causing the visual problem: for example, the eyes and the skin of a child with oculocutaneous albinism need protection from the sun as it is very sensitive to light. Clear explanations will help those who care for the child use the interventions recommended
  **Figure:** Teaching a young person with low vision how to use a telescope. SOUTH AFRICA
- The meaning of the distance and near visual acuity. For example, say: “Your child can now see 6/24 with the new spectacles. This means she can now read the blackboard from the front row in her classroom”
- Demonstrate to parents and teachers what the child struggled to do before receiving low vision interventions; use real activities such as reading from a school book. Show what they are able to do now that they are sitting near the window (for example) and have received spectacles and/or magnification. This will show parents and teachers the importance of following advice about lighting, wearing spectacles, and using any devices prescribed
- Explain any simple interventions that can help the child to use their vision better, e.g., sitting near the window, using a reading stand, ensuring there are contrasting lines on the stairs
- Explain how and when the child should use the magnifying device(s) prescribed and how to explain to peers how this device is helping them
- Most important: emphasise the need for regular follow-up!

## Working with older people

With older people, one should remember that most of them will have had good vision for most of their life and will have lived independently.

As people get older, it is harder to change habits and to learn new things. Patience and encouragement are very important. We must also be alert to the fact that older people are likely to have other impairments or conditions which can impact on the assessment as well as what is advised and the frequency of follow-up. The following may be present:

- Orthopaedic problems, such as an inability to bend fingers due to arthritis. This may make it difficult to use a hand-held magnifier
- Neurological problems, such as weakness of one arm, or tremors. These may also make hand-held magnifiers difficult to use
- Loss of hearing means that the individual will feel very isolated, and that the assessment, explanations, and counselling may take longer
- Neurological problems, such as dementia, may cause people to forget that they have a magnifier, or how to use it
- Mental health problems such as anxiety or depression are common in older people and may be made worse by a loss of vision. Such people may be too pre-occupied to explain what their problems are, or to concentrate and listen to what is being said to them.

Older people may have to take a range of medications. Ask about this when assessing them. Try to find ways to help them label bottles to ensure they take their medication as prescribed.

If transport is not an obstacle, do the assessment, prescribing, advice, and counselling in several separate visits. Suggest that they are accompanied by a younger relative or a carer.

**CASE STUDY 4**

**This 14-year-old boy** came with complaints of struggling to see the small print in his school textbook, which had reduced his reading speed considerably. He said that he was unable to find and pick up anything he dropped or misplaced. He also reported having difficulty in moving in unfamiliar areas and was not able to play with his friends in school. He had difficulty in identifying food and eating neatly.

After a complete low vision assessment, he was given distance vision spectacles. A14D stand magnifier along with a table lamp and reading stand helped him to read and do near work. He was also advised to use a torch at night and a cap to control glare during the day, and to use a dark-coloured lunch box to improve contrast. At a follow-up visit, he reported that the magnifier was comfortable for reading and that he could now read faster. Simple environmental modifications (the cap, lunch box, extra light, etc.) had improved his independence.
